# Wellness or medicine? Use and perception of Ayurveda in Germany: data from an online-representative cross-sectional study

**DOI:** 10.3389/fmed.2024.1408609

**Published:** 2024-05-22

**Authors:** Julia K. Schiele, Michael Jeitler, Andreas Michalsen, Elmar Stapelfeldt, Miriam Ortiz, Mike Sigl, Benno Brinkhaus, Manfred Wischnewsky, Christian S. Kessler

**Affiliations:** ^1^Institute of Social Medicine, Epidemiology and Health Economics, Charité - Universitätsmedizin Berlin, Corporate Member of Freie Universität Berlin and Humboldt-Universität zu Berlin, Berlin, Germany; ^2^Department of Pediatrics, Division of Oncology and Hematology, Charité - Universitätsmedizin Berlin, Corporate Member of Freie Universität Berlin and Humboldt-Universität zu Berlin, Berlin, Germany; ^3^Department of Internal and Nature-Based Therapies, Immanuel Hospital Berlin, Berlin, Germany; ^4^Institute for Cultural Studies, Humboldt-Universität zu Berlin, Berlin, Germany; ^5^Department of Mathematics and Computer Science, University Bremen, Bremen, Germany

**Keywords:** Ayurveda, survey, traditional medicine, complementary medicine, integrative medicine, alternative medicine, Germany, cross-sectional study

## Abstract

**Introduction:**

Ayurveda, South Asia’s largest and most relevant system of Traditional Medicine, holds a legal status akin to conventional Western medicine in India and elsewhere. There is an almost complete lack of data on the use of Ayurveda in Germany. The aim of this study was to investigate Ayurveda’s utilization patterns, entry points, and factors influencing its use and the perception of Ayurveda among the German population.

**Methods:**

Basis of this manuscript was an online-representative survey which involved 4,065 participants aged 18–75 about the use and acceptance of Traditional, Complementary and Integrative Medicine (TCIM) in Germany. The survey was conducted online using Computer Assisted Web Interview (CAWI) in 2022. The dataset was analyzed descriptively and inferentially.

**Results:**

Altogether 9.3% (*n* = 377) of all survey participants (*n* = 4,065) had already used Ayurveda somehow, either more often (1.7%) or at least once in a lifetime (7.6%). Responders associated Ayurveda primarily with Indian Medicine (27.7%) and wellness (18%). Commonly used Ayurvedic services included non-medical treatments at wellness resorts/spas (48.3%), in outpatient practices (27.1%), and hotels (23.6%). 30.2% of the participants believe in Ayurveda’s therapeutic potential. 76.7% of Ayurveda users find healthy nutrition important or very important. Nine predictors were found to classify Ayurveda users vs. non-users with spirituality and belief in Ayurveda’s therapeutic efficacy as the most relevant ones. Ayurveda seems to be primarily used by well-educated and female individuals, often from higher-income groups and with a rather modern social milieu-orientation.

**Conclusion:**

Study results suggest that about every tenth German citizen has used Ayurveda in the past and about one third believes in its therapeutic potential. Because Ayurvedic therapies are often not evidence-based, there is an urgent need to perform high quality randomized controlled trials to investigate potential effects and safety of Ayurveda and how evidence-based Ayurveda treatments can be integrated into the German healthcare system.

## 1 Introduction

Ayurveda is a comprehensive Whole Medical System (1, 2) characterized by a person-centered approach (Patient Centered Medicine, PCM) with system inherent diagnosis and treatment modalities, especially dealing with health promotion, recommendations on nutrition and disease prevention (3, 4). Ayurveda aims to shift focus from a disease-centered model to one centered on the well-being of each individual (5, 6). Regarding their PCM approaches, similarities exist between traditional systems of medicine like Ayurveda and modern approaches of predictive, preventive and personalized medicine. This renders Ayurveda not only “traditional,” but also potentially compatible with modern medicine (7–9). “Ayurgenomics” for example, a recently established field of research in South Asia, acts as a bridge between conventional genomics and Ayurveda, facilitating a deeper understanding of individual variations in response to Ayurvedic therapies across various diseases (10). Ayurveda provides comprehensive and cause-oriented traditional approaches for many chronic illnesses such as osteo- and rheumatoid arthritis, neurodegenerative diseases, kidney and liver diseases, irritable bowel syndrome, chronic inflammatory conditions, stress-related disorders, psychosomatic ailments or pain (11). Essentially in the realms of enhancing self-efficacy, salutogenesis, prevention, and healthy aging, Ayurveda offers patients and practitioners potentially valuable health-promoting opportunities (3, 11).

In India and some neighboring South Asian countries, Ayurveda is government-regulated, in India even by an independent ministry (AYUSH) (12), legally at par with conventional Western medicine. As a mainstream medicine it is providing health care opportunities in the most populous country worldwide with currently over 1.4 billion inhabitants. The importance of Ayurveda in Indian healthcare is reflected by the following figures: According to AYUSH, in India alone, more than 750,000 Ayurvedic physicians are officially registered; Ayurvedic medicine is systematically taught, practiced and supported by both AYUSH and union state governments in India in India in numerous universities and colleges universities and colleges (13). Altogether there are 495 Ayurveda colleges in India (13). Among them the university clinic “All India Institute of Ayurveda” (AIIA) (14) stands out as a public beacon-institute for clinical practice and research. Ayurvedic terminology, training and practice were recently standardized in WHO benchmark reports (15–17). In India, Ayurveda is currently (re-)gaining its own prominent place alongside conventional Western medicine, offering patients an integrative and multimodal approach to well-being and patient care (18). As such, in its region of origin, Ayurveda could even be seen (from the emic Indian perspective) as one form of “conventional” medicine in contrast to a predominantly European perception (of Ayurveda) as a foreign traditional medical system implemented either complementary or alternatively to conventional medicine.

The World Health Organization (WHO) describes Traditional Medicine like Ayurveda as *“the total sum of the knowledge, skills and practices indigenous (…) cultures have used (…) to maintain health and prevent, diagnose and treat (…) illness”* (19). WHO’s new vision for Traditional Medicine is the evidence-based integration of traditional medicine systems (TMS) into global healthcare. This is one goal of the first Global WHO Center for Traditional Medicine and the WHO Traditional Medicine Global Summit (20). In addition, the WHO highlights the economic dimension as a key argument in favor of TCIM systems like Ayurveda for cost-effective contributions for global health (20, 21). The WHO employs the term Traditional, Complementary, and Integrative Medicine (TCIM), or Traditional, Complementary, and Integrative Healthcare (TCIH) (22). For the sake of inclusivity and clarity, we adopt TCIM as a comprehensive umbrella term in this context. TM serves as a complement, integration, or alternative to conventional medicine (23, 24), represented by phrases such as ‘Complementary Medicine’, ‘Integrative Medicine’, and ‘Alternative Medicine’. These terms often overlap in content, and their distinctions can be blurred (25). The lack of precision is also evident in the diversity of definitions and differentiations among these methods. Historically, since the 1980s, the term ‘Complementary and Alternative Medicine’ (CAM), introduced by the National Institutes of Health (NIH), gained traction in the Anglo-American sphere. However, it has been supplanted by the modified term ‘Complementary and Integrative Medicine’ (CIM), which emphasizes the “integrative” aspect over “alternative,” underscoring the incorporation of evidence-based complementary medical practices into conventional treatment modalities (26–28). In Germany, therapeutic TCIM approaches, alongside conventional therapies, enjoy widespread societal acceptance (29, 30). In the past three decades, professional associations and organizations have attempted to bring more standardization and safety to Ayurvedic therapy and education in Germany and Europe. The establishment of the German Medical Doctors Association of Ayurvedic Medicine (Deutsche Ärztegesellschaft für Ayurveda-Medizin, DÄGAM e.V.) (31) in 2011 and the Ayurveda Umbrella Organization Germany (German: Ayurveda Dachverband Deutschland, ADAVED e.V.) (32) are indicative examples of the growing trend of professionalization of Ayurveda in Germany. These organizations, especially DÄGAM and ADAVED (32), are intensively involved in the development of training standards for various medical professions to promote quality assurance and are orientated toward the WHO benchmarks for Ayurveda (15, 16). DÄGAM, e.g., provides a quality certificate for Ayurveda training courses. The Association of European Ayurveda Therapists (Verband Europäischer Ayurveda-Therapeuten e.V., VEAT) (33), the Academic Society of Indian Medicine (Akademische Fachgesellschaft Indische Medizin e.V., AFGIM) (34, 35), the Indian Society for Ayurveda Germany (Indische Fachgesellschaft für Ayurveda Deutschland, IFAD e.V.) and the German Society for Ayurveda (Deutsche Gesellschaft für Ayurveda, DGA e.V.) (36) also promote medical Ayurveda in Europe. However, there is a contrast between these endeavors as well as the therapeutic potential for patients and the fact that there is still *de facto* no reimbursement by statutory health insurances or any kind of official recognition of Ayurveda in Germany. In principle however, any medical doctor (MD) or alternative practitioner (German: Heilpraktiker) (37) can practice Ayurveda independently within the scope of their professional medical licenses in Germany. In comparison, in Switzerland it is possible to get a federal diploma in Ayurvedic therapy and there are different trainings for physicians or alternative practitioners (38). However, unlike qualification possibilities for the aforementioned, acupuncture or Traditional European Medicine (German: Naturheilkunde/Naturheilverfahren) that exist in Germany, there is no explicit foundation for medical care with Ayurveda methods and billing of Ayurvedic services. In general, the so-called prevention paragraph [§20 Sozialgesetzbuch (SGB) Fifth Book (V)] in Germany supports public health promotion and preventive measures by statutory health insurance funds (39). This opens potential possibilities for Ayurveda to play a role in (reimbursable) public health care, like other already integrated traditional medicine options, such as acupuncture for some indications.

While some previous representative studies in Germany indicated a high usage of TCIM in Germany, there is no such data on Ayurveda. In 2014 a project examined the use of TCIM in Germany, but it is likely that the data has become outdated (40). Despite an anticipated growing interest in Ayurveda as a medical approach, there remains a notable scarcity of robust representative survey data regarding its overall use and user perception of it in Germany (41). This publication aims to provide insights into aspects such as utilization patterns, application contexts, points of entry, and perceptions regarding its efficacy. This work also intends to explore influences of social backgrounds and individual belief systems that might contribute to the use of Ayurveda.

## 2 Materials and methods

The Charité University Outpatient Clinic for Complementary and Integrative Medicine at Immanuel Hospital Berlin and the Institute of Social Medicine, Epidemiology and Health Economics of Charité—Universitätsmedizin Berlin conducted an online-representative survey about the use and acceptance of Traditional, Complementary and Integrative Medicine (TCIM) from September to October 2022. The study design has been described in detail elsewhere (42). In short, the overall research project encompassed both a representative cross-sectional survey and a qualitative study. This study is based on a cross-sectional online survey using Computer Assisted Web Interview (CAWI) among the German-speaking residential population aged 18–75 years, with a total sample size of 4,065 individuals. The study was approved by the Charité Ethics Committee and registered with ClinicalTrials.gov (NCT05530720). The online panel adheres to the international standard ISO 26362, ensuring that the quality of online sampling is monitored and certified. The procedures for data collection, processing, and storage followed internationally recognized guidelines for clinical studies, including the Declaration of Helsinki and ICH-GCP (International Council for Harmonization of Technical Requirements for Pharmaceuticals for Human Use). In addition, the ethical principles of the accompanying sociological research were followed.

The comprehensive questionnaire used in this study covered a wide range of topics: sociodemographic data, use of TCIM, attitudes toward TCIM, diagnoses for which TCIM was used, importance and familiarity with terms, the role of TCIM in the context of the Covid-19 pandemic, nutrition, Ayurveda, attitude and behavior, Sinus milieu indicator^®^ and the EQ-5D-5L quality of life questionnaire. It contained a module of five items on Ayurveda (Supplementary material 8).

The sample selection was quota-based and structured to ensure representation across different age groups, genders, levels of education, and geographic regions. These quota specifications were established following the methodology standard set by the best4planning (B4P) study, which is known for drawing representative samples (43). The B4P study itself was based on a random selection of over 30,000 individuals. Quota control was employed to address socio-demographic disparities when compared to the general population.

### 2.1 Statistical analysis

Continuous variables are given as mean ± standard deviation (SD), or median and interquartile range, and categorical variables as frequencies (percentages). Normality of distribution was assessed by the Shapiro–Wilk test. Continuous variables were tested for differences with the Mann–Whitney U test or the Wilcoxon signed-rank test, and categorical variables with the Pearson’s χ^2^ or the Fisher’s exact test. Crosstab analysis revealed patterns, correlations, and trends among categorical (nominal or ordinal) variables. Decision trees (DTs) were used as a non-parametric supervised learning method for classification and regression. The goal is to create a model that predicts the value of a target variable by learning simple decision rules inferred from the data features. We applied Exhausted Chi-squared Automatic Interaction Detection (Exhausted CHAID) and Classification and Regression Trees (CART). Variable importance for predictors in decision trees, such as CHAID and CART, are based on measures of sensitivity. The sensitivity of a variable is a measure of the amount of output variance that is removed when we learn the true value of the predictor. Normalized importance is simply the importance values divided by the largest importance values and expressed as percentages. Variable (normalized) importance as measured by the sensitivity analysis does not predict the order in which predictors appear in the decision tree. In this study, we do not report demographic weighting (age, gender, education, federal state, and city size). The bias would only be minimally reduced, and we found no statistically significant differences between weighted and unweighted results. All statistical analyses were performed using R (R Foundation, version 4.3) and SPSS (IBM^®^ SPSS^®^ Statistics, version 29).

## 3 Results

The survey took place between September and October 2022, with 41,011 invitations distributed. Of these, 8,821 individuals initiated the survey, yielding a response rate of 21.5%. Based on the exclusion criteria, such as cases where participants had reviewed the study information but did not provide consent or lacked age information, 453 cases were excluded. Furthermore, 2,845 participants were removed as they had already been assigned to filled quotas. Exactly 1,000 individuals discontinued their participation and were consequently excluded from the analysis. Ultimately, 4,505 respondents successfully completed the questionnaire. During the quality assessment process, 18 participants were excluded due to discrepancies identified in the sinus milieu indicator. Subsequently, an additional 295 participants were excluded for quality-related issues, including anomalous open responses and discrepancies in quality variables. The final data set comprised 4,210 participants. In order not to compromise online representativeness due to the upper age limit of 80 years, this was reduced to 75 years. Thus, the final population-representative data set for the age group 18–75 years comprises a total of 4,065 participants.

Among all participants (*n* = 4,065, aged 18–75) 51.7% were female, 47.9% were male and 0.4% diverse. 42.5% had a higher education (high school diploma, doctorate, university degree) and more than half of the study population had completed primary and secondary school (56.8%). Approximately half of the participants (54.3%) had a net household income between 2,000 and 5,000€, while 7.5% had an income above this amount and 38.2% had an income below this amount. The socio-demographic characteristics can be found in detail in the main publication (42). Among the users of Ayurveda services (*n* = 377) 61% were female, the majority earning between 2000 and 5,000 € per month (60.7%). 53.2% hold a higher school diploma (Table 1). Regarding the sociodemographic characteristics of Ayurveda services use, the parameters age (*p* = 0.007), gender (*p* < 0.001), net monthly household income (*p* = 0.005), education (*p* < 0.001), nutrition (*p* < 0.001), spirituality (*p* < 0.001) and attitude toward TCIM (*p* < 0.001) differed significantly in comparison to non-Ayurveda users (Table 1).

**Table 1:** Basic characteristics.

		Total	Ayurvedic services used		*p*-value
			Yes	No *or* I do not know	
		4,065	377 (9.3)	3,688 (90.7)	
Age, years		49.3 ± 15.8	47.2 ± 15.8	49.5 ± 15.8	0.007
Gender	male	1,947 (47.9)	146 (38.7)	1,801 (48.8)	< 0.001
	female	2,101 (51.7)	230 (61.0)	1,871 (50.7)	
	diverse	17 (0.4)	1 (0.3)	16 (0.4)	
Net monthly household income	≤ 2000€	1,552 (38.2)	115 (30.5)	1,437 (39.0)	0.005
	2000–5,000€	2,208 (54.3)	229 (60.7)	1,979 (53.7)	
	> 5,000€	305 (7.5)	33 (8.8)	272 (7.4)	
Education	(Yet) no general school-leaving certificate	30 (0.7)	1 (0.3)	29 (0.8)	< 0.001
	Primary / secondary school	2,309 (56.8)	176 (46.7)	2,133 (57.8)	
	A-levels (technical) university entrance qualification without studies, Studies (university, college, university of applied sciences, polytechnic, PhD^2^)	1,726 (42.5)	200 (53.1)	1,526 (41.4)	
Nutrition	less important to completely unimportant	1,530 (37.6)	88 (23.3)	1,442 (39.1)	< 0.001
	important / very important	2,535 (62.4)	289 (76.7)	2,246 (60.9)	
Sinus Main Milieus^®^	Society’s Leading Milieus	1,456 (35.8)	149 (39.5)	1,307 (35.4)	0.058
	Modern Mainstream	1,135 (27.9)	95 (25.2)	1,040 (28.2)	
	Milieus of the Future	742 (18.3)	79 (21.0)	663 (18.0)	
	Traditional Mainstream	732 (18.0)	54 (14.3)	678 (18.4)	
Spirituality	yes	896 (22.0)	190 (50.4)	706 (19.1)	< 0.001
	no	3,169 (78.0)	187 (49.6)	2,982 (80.9)	
Attitude towards conventional medicine	mostly positive, very positive	2,565 (63.1)	238 (63.1)	2,327 (63.1)	0.976
	neutral	1,187 (29.2)	109 (28.9)	1,078 (29.2)	
	mostly negative, very negative, do not know	313 (7.7)	30 (8.0)	283 (7.7)	
Attitude towards TCIM^1^	mostly positive, very positive	2,112 (52.0)	277 (73.5)	1,835 (49.8)	< 0.001
	neutral	1,519 (37.4)	87 (23.1)	1,432 (38.8)	
	mostly negative, very negative, do not know	434 (10.7)	13 (3.4)	421 (11.4)	

^1^TCIM: Traditional, Complementary and Integrative Medicine, ^2^PhD: Philosophical Doctorate.

### 3.1 Use and associations with Ayurveda, entry points and services used

**Figure 1:**
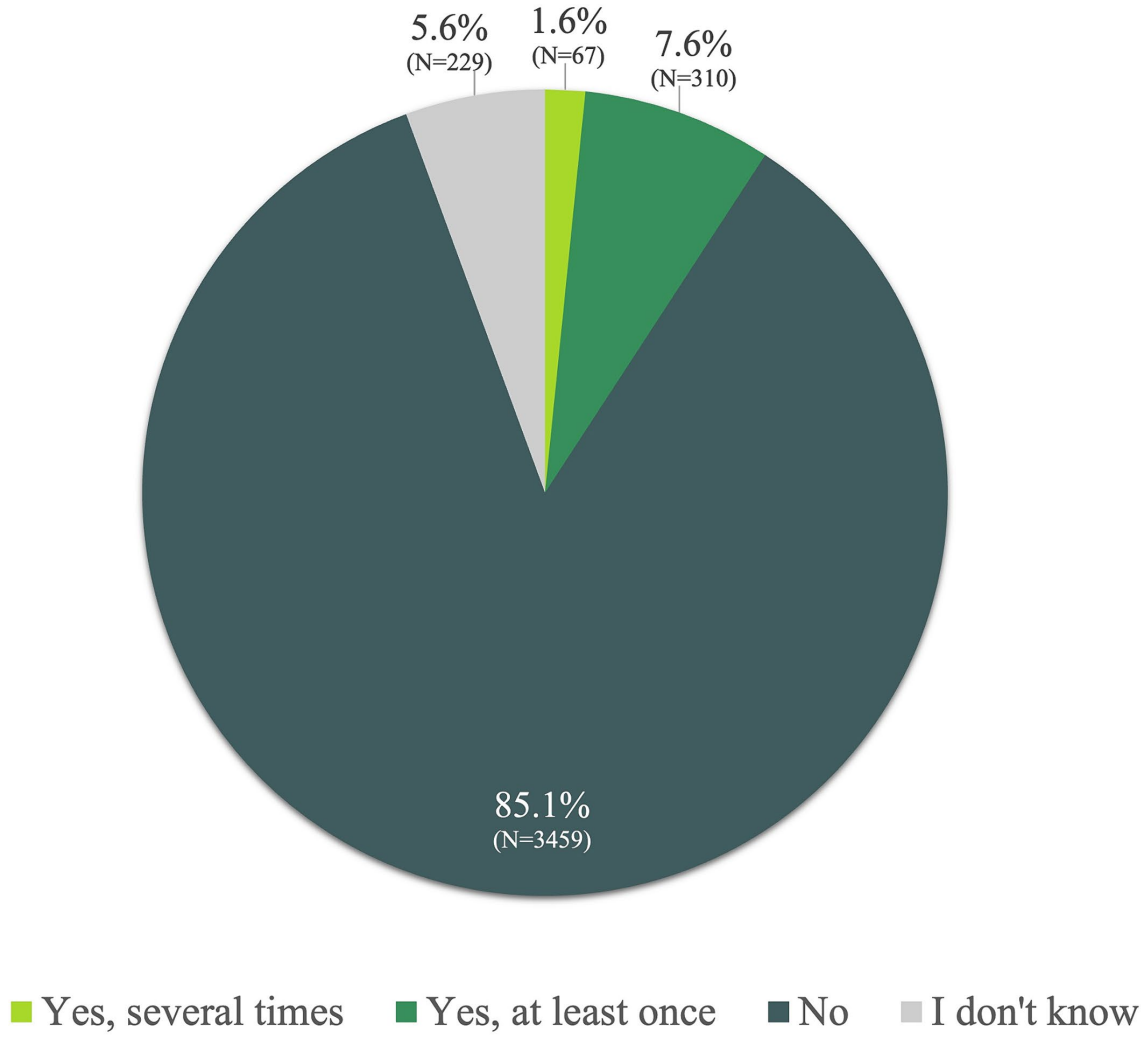
Have you ever used Ayurvedic treatments yourself?

A small percentage (1.6%) of the whole study population reported having used Ayurveda services more than once, while 7.6% had used them at least once. The majority (85.1%) had never utilized Ayurveda services (Figure 1).

**Figure 2:**
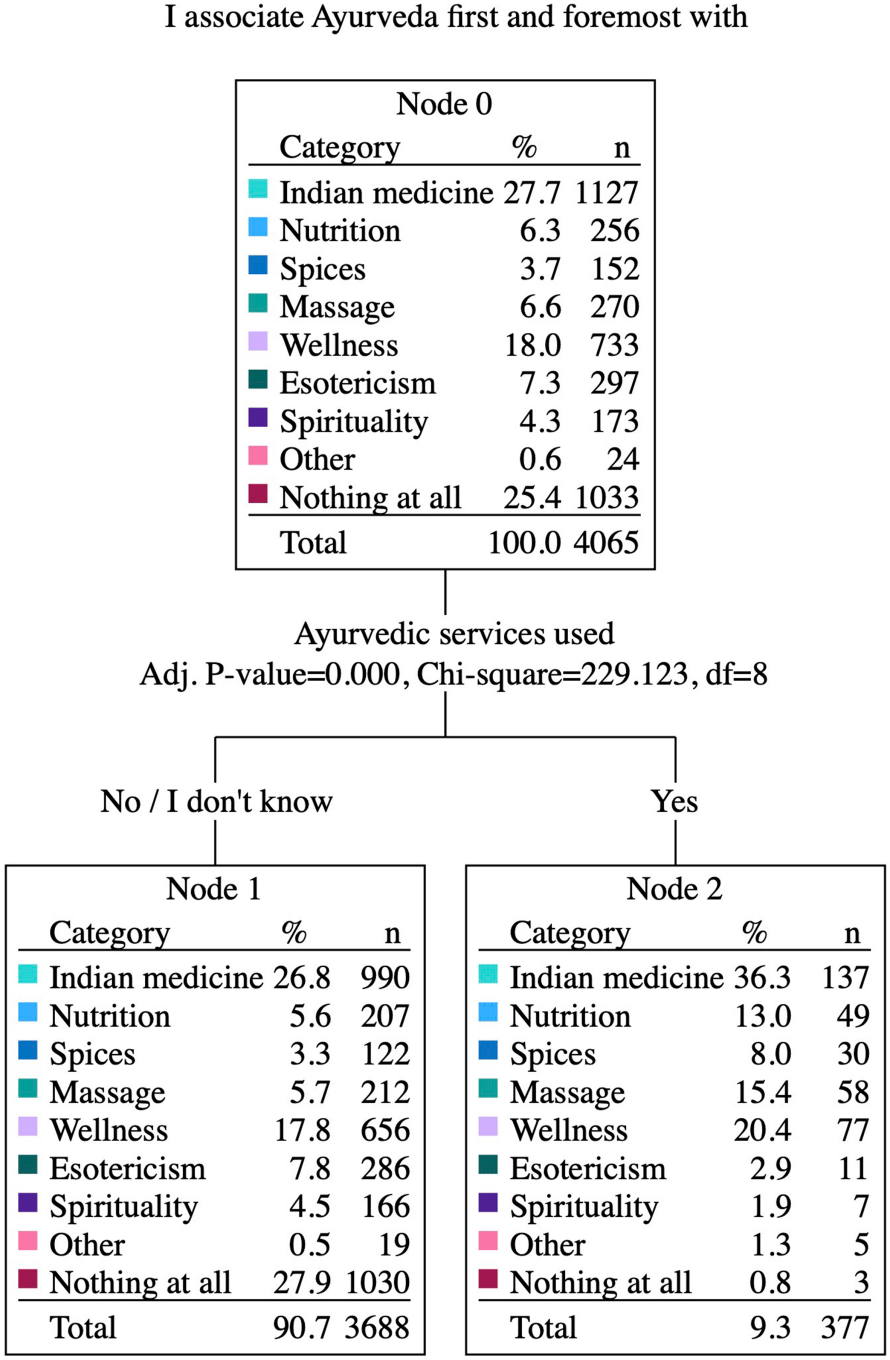
I associate Ayurveda first and foremost with...

Participants’ associations with Ayurveda predominantly include Indian Medicine (27.7%) or wellness (18%), a large group (25.4%) had a lack of specific associations. Esoteric concepts, on the other hand, were less frequently linked, with only 7.3% mentioning them. Keywords like massage, nutrition, spirituality, or spices were rated lower than 7%. Among Ayurveda users (*n* = 377), the most frequent association was also Indian medicine (36.3%), followed by wellness (20.4%), massage (15.4%) and nutrition (13%). Among non-Ayurveda users the most rated categories were Indian medicine (26.8%), followed by wellness (17.8%), esotericism (7.8%) and massage (5.7%). The association with Ayurveda differs highly significantly between the participants with or without Ayurveda services (*p* < 0.001; Figure 2).

The 377 Ayurveda users stated that they had used a total of 666 Ayurveda services. 42.2% had used Ayurveda products, 39.8% non-medical Ayurveda treatments, 24.9% had had Ayurveda nutritional consultation and 22.3% had experienced medical Ayurvedic treatments, another 22.3% had undergone Ayurveda lifestyle consultation (Table 2).

**Table 2:** Which Ayurveda services did you use? (multiple response).

	Responses		Percent of Ayurveda users
	N	percent	
Ayurveda products (e.g., food, cosmetics, food supplements…)	159	23.9%	42.2%
Non-medical Ayurveda treatments (e.g., by alternative practitioners or physiotherapists)	150	22.5%	39.8%
Ayurveda nutritional consultation	94	14.1%	24.9%
Ayurveda lifestyle consultation	84	12.6%	22.3%
Medical Ayurvedic treatments	84	12.6%	22.3%
Infotainment (TV, movies, internet)	36	5.4%	9.5%
Ayurveda-Training	34	5.1%	9.0%
Other	25	3.8%	6.6%
Total	666	100.0%	176.7%

**Figure 3:**
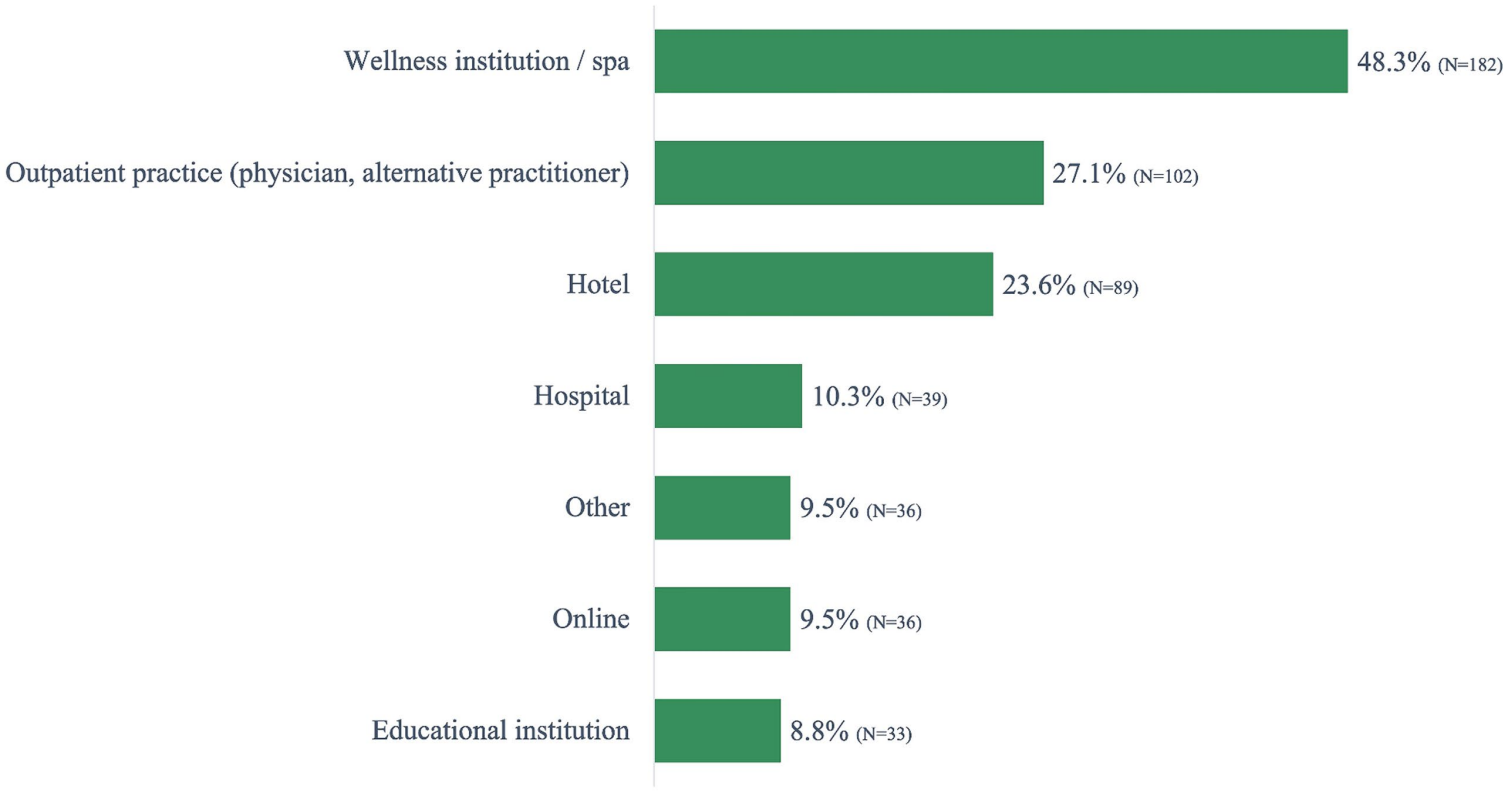
Where have you used Ayurveda services? (multiple response).

The Ayurveda users reported accessing Ayurveda services most frequently at wellness resorts (spa; 48.3%), in an outpatient practice (physician or alternative practitioner; 27.1%), and hotels (23.6%). A smaller percentage (9.5% or lower) experienced Ayurveda services online, via educational institutions or other (Figure 3).

### 3.2 Perception of therapeutic efficacy

**Figure 4:**
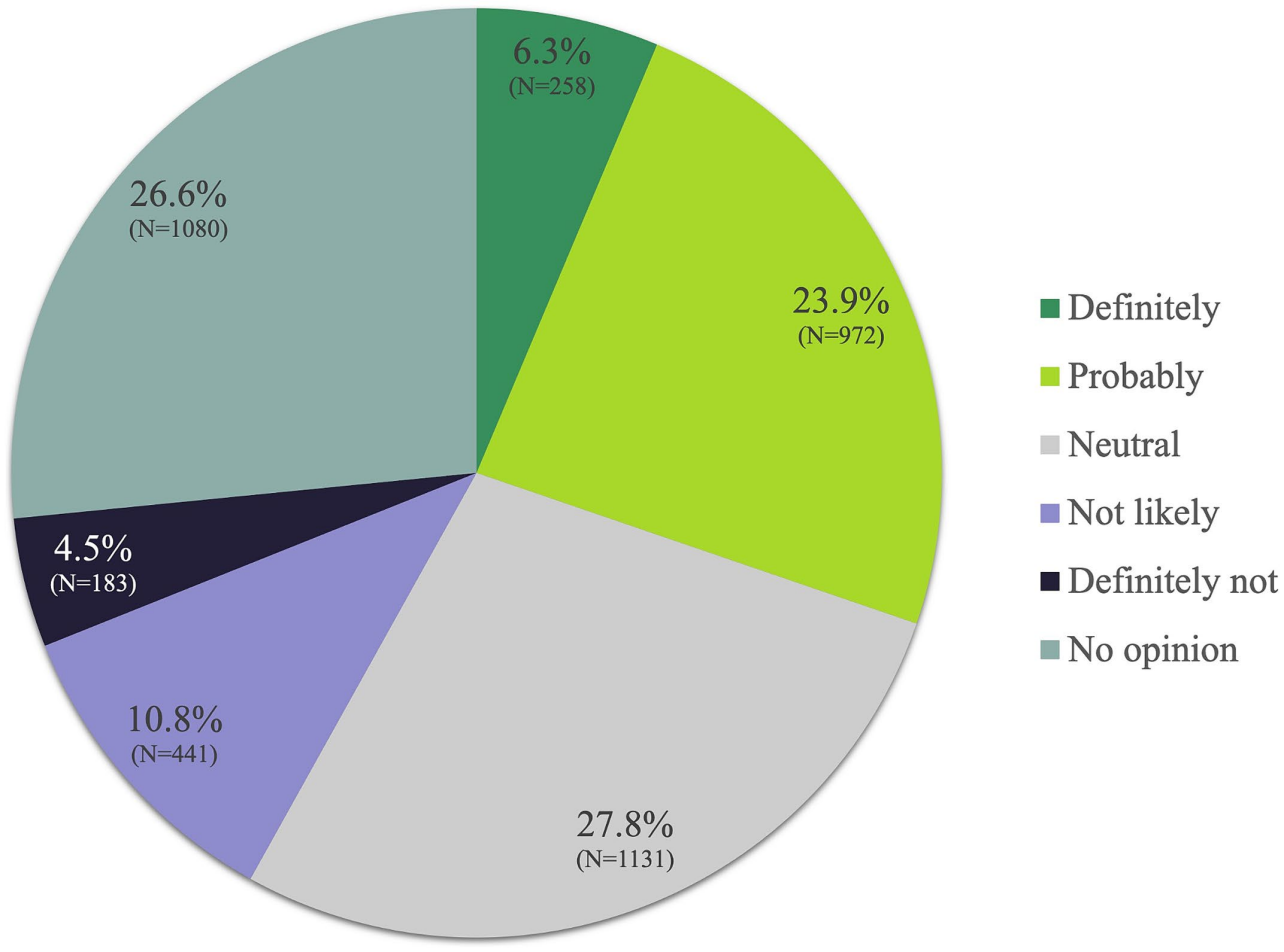
Do you think that Ayurveda has any medical therapeutic benefits?

6.3% of all participants “definitely” believed in the medical therapeutic benefits of Ayurveda and 23.9% responded “probably.” Additionally, 27.8% responded with “neutral” and 26.6% had “no opinion” on it, while 10.8% thought of potential benefits as “not likely” to exist or materialize. Another 4.5% believed that Ayurveda “definitely” had no benefits (Figure 4).

Significantly (*p* < 0.001) more participants who had already used Ayurveda believed in its therapeutic benefits than participants who had no previous experience with Ayurveda. Thus, Ayurveda users answered “definitely” (28.4%) or “probably” (40.1%) in comparison to non-Ayurveda users (4.1% or and 22.3%, respectively; Supplementary Figure 1).

Looking at subgroups who believed that Ayurveda had major therapeutical benefits, 47.4% of the participants who had a “mostly positive” or “very positive” overall attitude toward TCIM also rated the therapeutic benefits of Ayurveda as positive (definitely/probably). If these participants in addition stated that they were spiritual and older than 20 years, then the percentage of these participants who viewed the therapeutic benefits of Ayurveda positively (definitely/probably) increased to 68.1%. Conversely, if the participants’ global attitude toward TCIM was negative (mostly or very negative) or if they said they “did not know,” then only 7.6% had a positive attitude toward the benefits of Ayurveda. If these participants also stated that they were not spiritual and younger than 40 years, then this percentage was reduced to 5.9%. Further details are provided in Supplementary Figures 2A–C.

### 3.3 Ayurveda and spirituality

**Figure 5:**
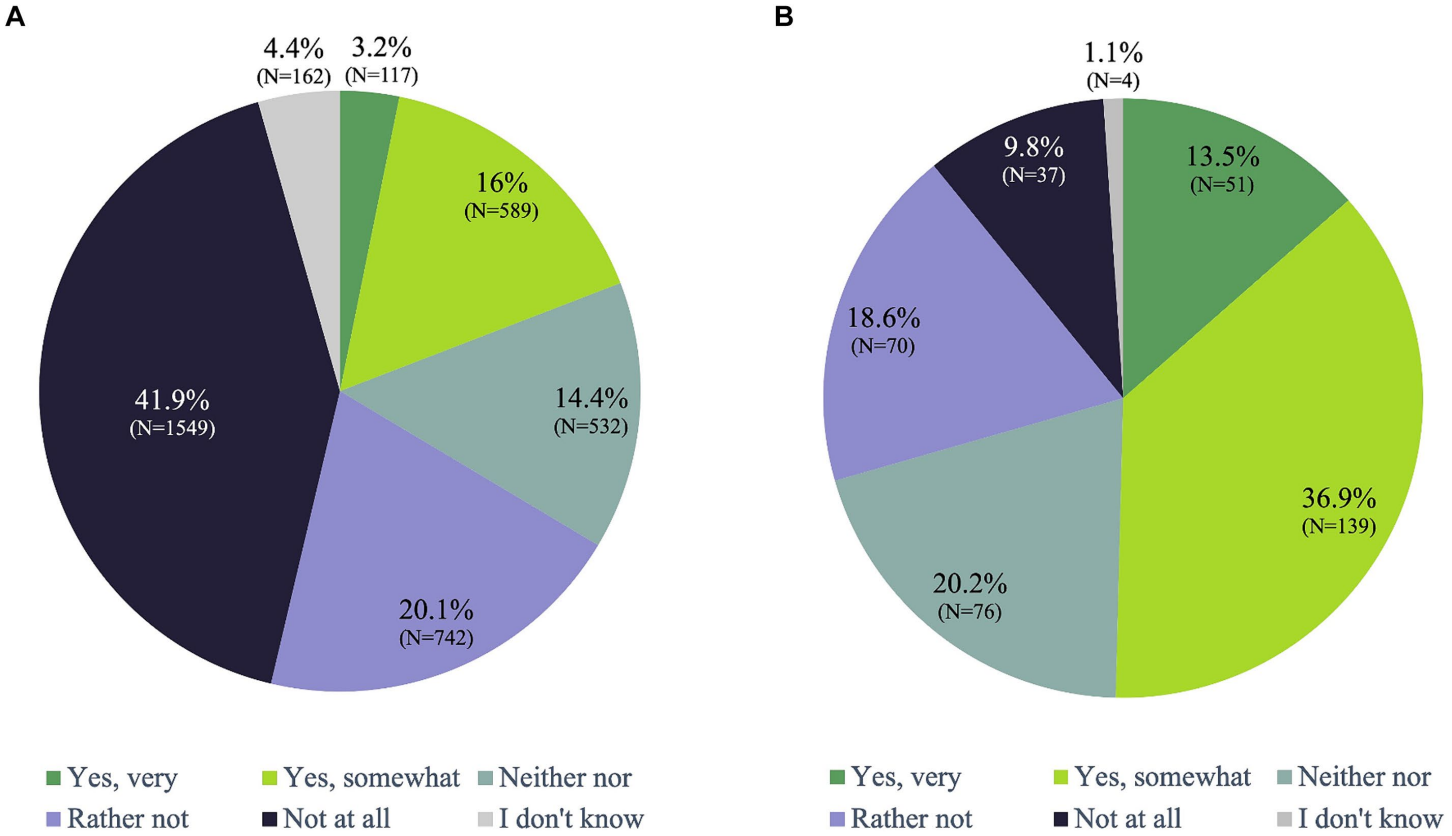
Would you describe yourself as spiritual? **(A)** Non-Ayurveda-Users (N=3688, 90.7%), **(B)** Ayurveda-Users (N=377, 9.3%).

In the total population, 17.9% considered themselves somewhat spiritual and 4.1% as very spiritual, while 38.9% claimed they are not at all spiritual (Supplementary Figure 3). When examining the 9.3% participants who used Ayurveda services, 50.4% were identified as very (13.5%) or somewhat spiritual (36.9%) and 9.8% as not spiritual (Figure 5B). Conversely, among the 90.7% participants who had not previously used Ayurveda services, 19.2% described themselves as very (3.2%) or somewhat (16.0%) spiritual and 41.9% as non-spiritual. Further details in Figures 5A,B.

Moreover, if we compare the religious affiliation, we get a significant difference (*p* < 0.001) between Ayurveda users and Ayurveda non-users (Supplementary Figure 4).

### 3.4 Classification of Ayurveda users and Ayurveda non-users

**Figure 6:**
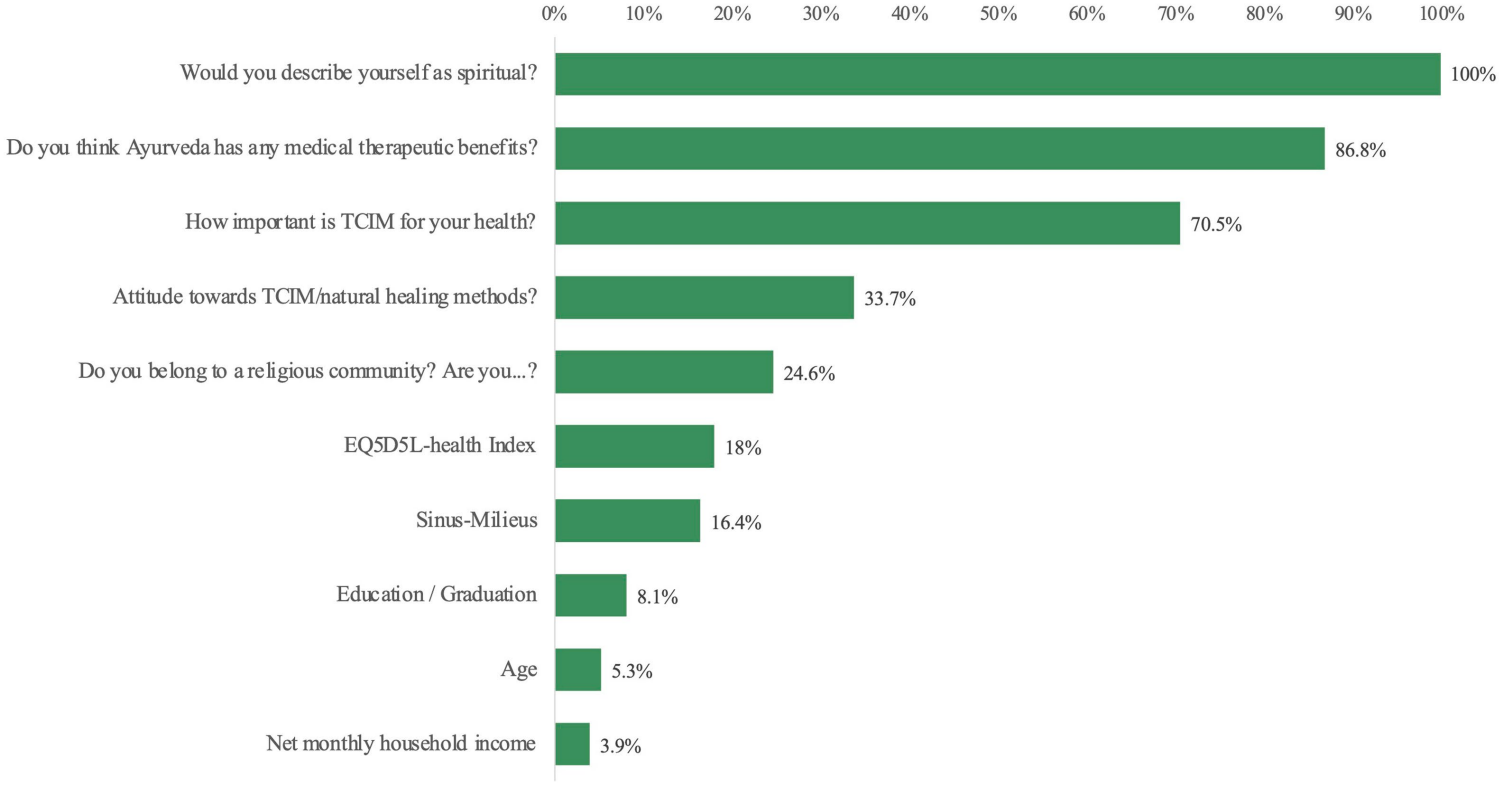
Order of normalized importance expressed as percentages to classify Ayurveda users and Ayurveda non-users.

Ayurveda users and Ayurveda non-users can be classified using a set of rules or equivalently by decision trees based on nine predictors with an accuracy over 90%. These predictors are in descending order of importance (Figure 6).

**Figure 7:**
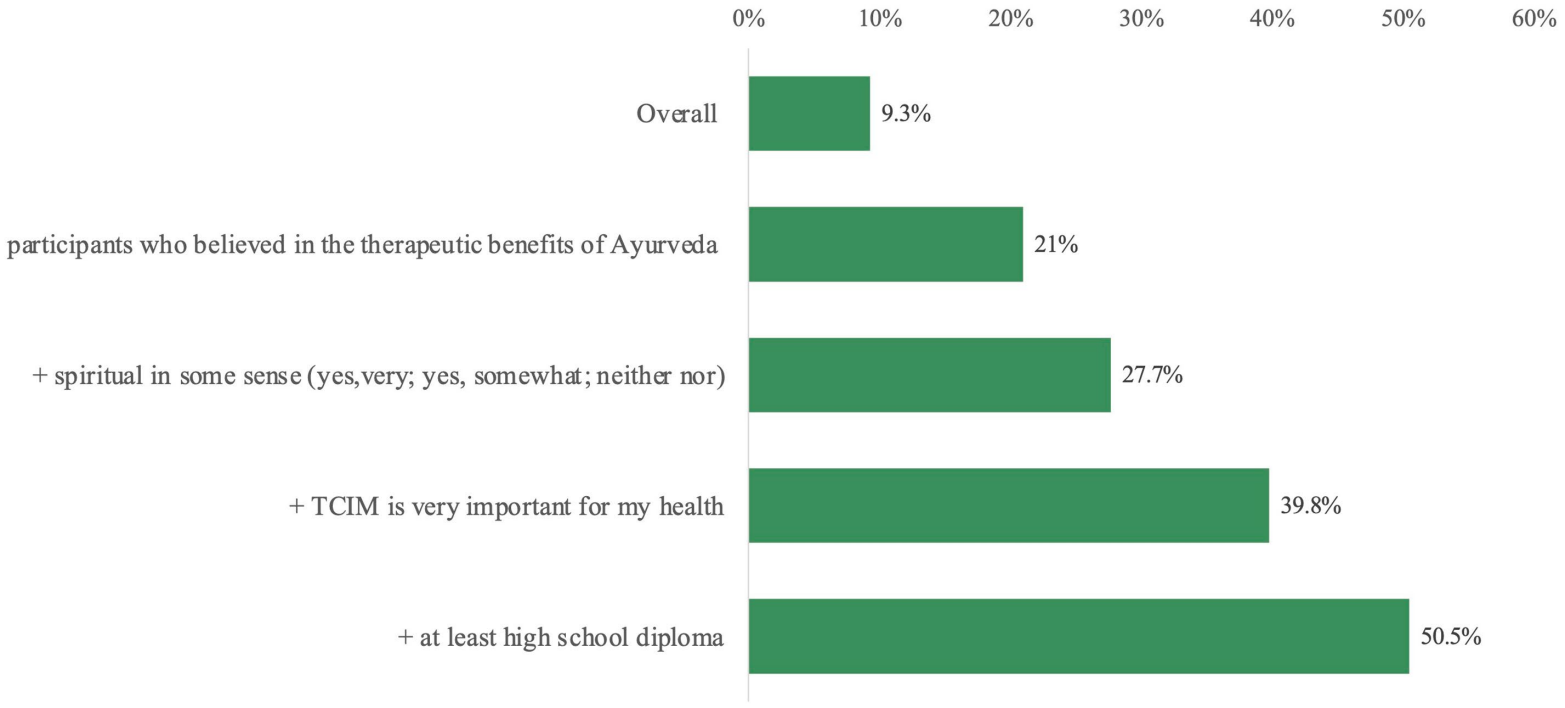
Increasing percentage of Ayurveda users within the total population.

Other possible predictors like “gender” (normalized importance 0.5%) or “global attitude to TCIM” (1.7%) are no longer significant in this multivariate model in contrast to univariate analyses. The following example from this decision tree shows the increasing percentage of Ayurveda users by gradually adding various predictors (Figure 7). Further details of this decision tree are shown in the Supplementary Figure 5.

### 3.5 Ayurveda users and healthy nutrition

76.7% of Ayurveda users found healthy nutrition important or very important. This proportion is significantly (*p* < 0.001) higher than in comparison to non-Ayurveda users (Supplementary Figure 6). There is also a significant (*p* < 0.001) difference in the choice of diet depending on the use of Ayurvedic services (Supplementary Figure 7). 21.5% of the Ayurveda-users characterized their nutrition as vegetarian, vegan or raw food-vegan/based. 35.3% characterized themselves as flexitarians.

### 3.6 Distribution of Sinus Milieus^®^ and Sinus Main Milieus^®^ in Ayurveda users

**Figure 8:**
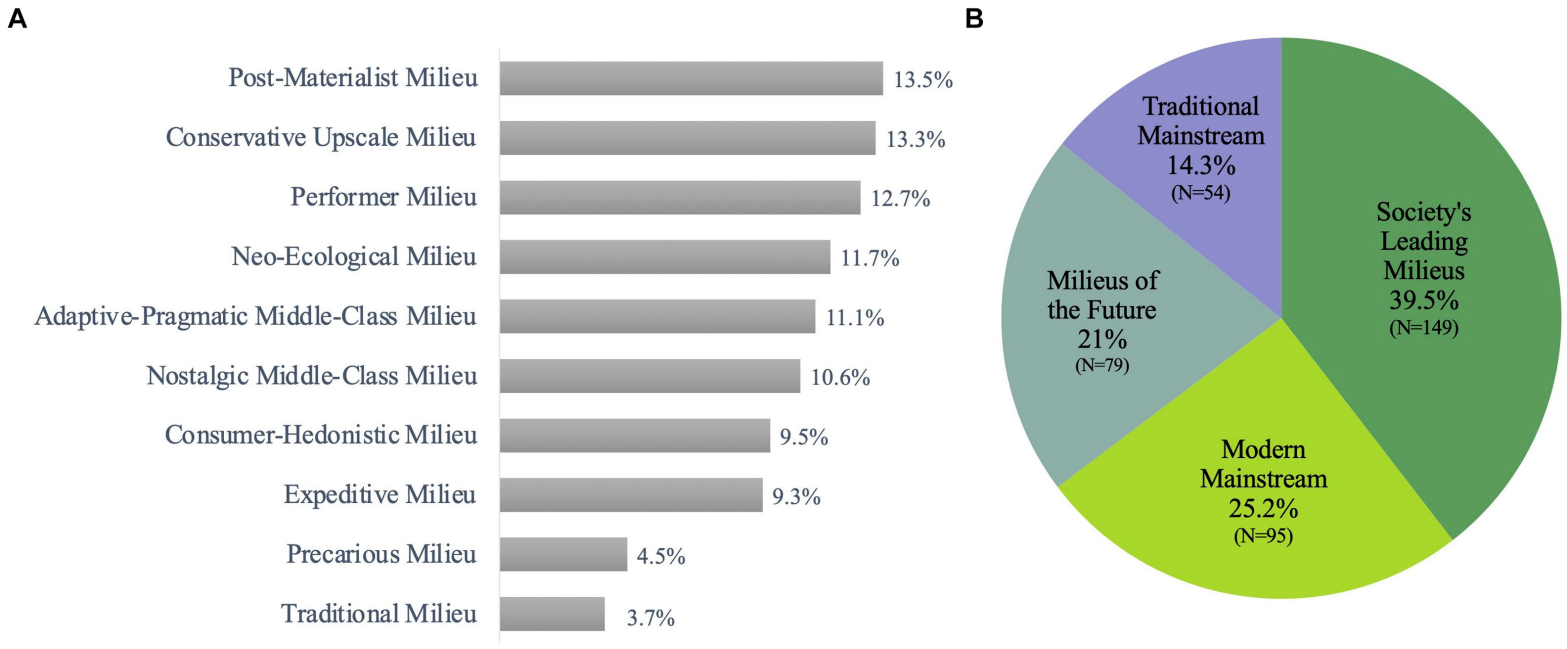
Distribuation of **(A)**: Sinus Milieus^®^ and **(B)**: Sinus Main Milieus^®^ in Ayurveda users.

Sinus-Milieus^®^ (available for more than 50 countries) are a social model, which summarizes participants with similar values, a similar lifestyle and a comparable social situation in groups of “like-minded people.” The transitions between the milieus are fluid. The Sinus-Milieus^®^ are defined by the social situation (ranging from low to high) and the value orientation (ranging from traditional to postmodern) (44). 39.5% of the Ayurveda users belonged to the Society’s Leading Milieus which consist mostly of following three milieus: Post-Materialist (13.5%), Conservative-Upscale (13.3%) and Performer (12.7%). 25.2% belonged to the Modern Mainstream [Adaptive-Pragmatic Middle Class (11.1%), Consumer-Hedonistic (9.5) and Precarious (4.5%)]. Only 3.7% of Ayurveda users came from a traditional milieu (Figures 8A,B).

The distribution of the Sinus Milieus^®^ differed significantly (*p* = 0.001) between Ayurveda users and Ayurveda non-users. The distribution of Sinus Main Milieus^®^, on the other hand, is borderline between both groups (*p* = 0.058).

## 4 Discussion

In this online-representative cross-sectional study involving 4.065 residents in Germany, almost about one in 10 Germans had already used some as Ayurveda identified services. The majority associated Ayurveda with Indian medicine, while almost the same number of participants had no specific associations. Popular Ayurveda services included treatments from alternative practitioners, Ayurvedic products, nutritional or lifestyle counseling and medical treatments, with spa or wellness services providing entry points. Nearly one third of the participants believed in Ayurveda’s therapeutic potential. The belief in therapeutic benefits of Ayurveda were fostered by parameters such as a positive attitude toward TCIM and spirituality. Ayurveda was primarily used by well-educated, female individuals, aged between 20 and 40 years, with interest in (vegetarian/vegan) nutrition, often from higher-income groups and with Modern Mainstream or Society’s Leading Milieu association. This could correspond to the profile of a “typical” TCIM user in the Western world (45, 46).

### 4.1 Strengths and limitations

This is the first study on the use and perception of Ayurveda in Germany including a large study population. Limitations of the study include a relatively low response rate at 21.5%, raising minor concerns about the applicability of the findings to a broader population. To enhance generalizability, the data utilized for analysis underwent weighting based on factors such as age, gender, education, federal state, and city size (42). However, no statistically significant differences between the weighted and unweighted data could be found, which suggests a sufficient quota system (42).

The phenomenon of low response in surveys is multifaceted and warrants thorough examination. Research has shed light on various factors that contribute to low response rates. For instance, Dillman et al. emphasized the impact of survey design and question quality on participant willingness to engage (47). Additionally, Singer and Kulka highlighted the significance of adequate incentives in boosting response rates (48). Demographic characteristics also play a role with factors such as age, education level, and employment status influencing survey participation (49). These findings underscore the importance of conducting a comprehensive analysis of factors influencing response rates to ensure accurate interpretation of survey results and to inform strategies for improvement. The study utilized an online access panel for surveying, chosen for its high-quality standards in participant selection and maintenance, as well as the implementation of a quota system (50). This approach ensures population-representative insights into the utilization and acceptance of TCIM in the German population. The online approach was selected due to the sensitivity of personal health-related questions. It is important to note that the use of an access panel led to the exclusion of certain populations, like those without online access or with low online affinity. However, this exclusion is not unique to access panels or online surveys in general. The potential under-representation of older people in this study had only a minor impact given the lower likelihood of them having internet access, as this age group was excluded from the analysis sample.

The study provide interesting data on Ayurveda use in Germany, however a limitation was that we did not ask specific details, e.g., regarding the types of Ayurvedic treatments and therapies used by participants. Without this information, it is challenging to determine which Ayurvedic interventions are more or less popular, limiting the insights into the aspects of Ayurveda that are in demand. While the survey mentions common locations for accessing Ayurvedic services, it does not provide a detailed breakdown of the preferences for these locations. Understanding why individuals choose one location over another could provide further context. These and other detailed questions would help us to better understand Ayurveda user demands in Germany. The data did also not contain information about which health conditions or diseases participants sought Ayurvedic treatments for. Understanding the specific health issues for which Ayurveda is being utilized in Germany could offer valuable insights into its effects and potential areas for further research. Moreover, side effects of Ayurveda were not asked. However, these limitations of our study can be addressed in future cross-sectional studies.

Finally, this data-set from a cross-sectional survey does not allow any causal statements. Also, the survey does not offer an in-depth comparison between Ayurveda and other traditional or complementary healthcare options, making it difficult to assess its relative popularity or effects. Those limitations highlight the need for more comprehensive and specific research to fully understand the role and efficacy as well as effectiveness of Ayurveda in the German context.

### 4.2 Discussion points

Ayurvedic interventions used in wellness resorts/spa facilities could presumably reflect the pattern that health-conscious people without an acute medical need are more likely to use low-threshold entry options and use Ayurveda for health-maintenance, prevention and well-being. While the use of Ayurveda as a wellness service could be a way to reach a wider audience, it could serve the medical visibility and credibility of Ayurveda to differentiate these services from explicit medical and therapeutic Ayurveda-options for specific health needs (51).

Almost one-third of the population (30.2%) believes in Ayurveda’s therapeutic potential, while an almost equivalent portion remains neutral or has no opinion on this. Moreover, participants which used Ayurveda in the past rated its potential benefit considerably higher. Scientific evidence in the field of Ayurveda is still at an infant stage in Germany and the European Union; only few methodologically high-quality trials which proof to live up to high (−quality) standards concerning the implemented methods were published so far (52–55). More methodologically high-quality research, professional communication with the public and public support (e.g., governmental, financial) would be necessary to define potential roles of Ayurveda as a complementary treatment option within the German health care system. Overall, there is a need for more clinical evidence, particularly effectiveness studies, to collect real-world data on Ayurveda interventions outside its countries of origin (56). Although available studies show promising results for Ayurveda in the field of clinical research for some diagnoses, the current state of research is still insufficient, especially against the background of the growing utilization of Ayurvedic services in Germany and the EU. Foremost, more high-quality clinical research is needed at German and European universities to be able to answer questions about the effectiveness of Ayurvedic therapies under local conditions. In this context, AYUSH exchange programs with foreign universities to promote Ayurveda, yoga and other traditional Indian medicine could be a relevant building block for the further development of the academic infrastructure required for this (12).

Spirituality, which appears to be associated with Ayurveda use, indicated by the fact that half of Ayurveda users appears to have a spiritual attitude (57, 58) is notable and requires further attention. Especially as the conception of “spirituality” may vary enormously on a social cultural level between the cultures of origin (India/Germany) and even within the respective milieus. The connection between Ayurveda and spirituality can be traced back to the close historical relationship between Ayurveda and Buddhism, Hinduism and Indian culture in general (59, 60), first described in the Caraka Samhita [see Gupta (61); Caraka Saṃhitā Śārīrasthāna chapters 1 and 5 (62)], which is one of the oldest systematic text collections of on Ayurveda. Previous publications explored the role of religion and spirituality in medical contexts, emphasizing that these elements may influence attitudes and choices regarding the use of Ayurvedic healthcare services. Spirituality plays a crucial role in how Ayurveda is perceived, and in India it coexists with modern medicine (63–66). In a paper of our working group associations were identified between individuals’ religious or spiritual affiliations and their choices to either provide or seek access to Ayurveda (67). Importantly, the utilization of Ayurveda did not preclude concurrent use of both modern medicine and TCIM (67). Also, in combination with yoga, Ayurveda has a long tradition in India of mind (−fullness) training, breath work or ethical-philosophical recommendations for everyday life, which also has found a “mainstream-compatible approach” e.g., into fitness- and yoga-studios (11, 68). In a systematic review by Jeserich et al. on the correlation between religion and spirituality (R/S) and the sense of coherence (SOC), a connection was found between R/S and mental health (64). Relevant effect sizes were found in relation to the potential resource of spirituality. These relationships with a sense of coherence, which is an important indicator of the capacity of resilient coping with difficult situations (such as coping with illness), was stronger the more semantically open and non-institutional the spiritual belief system was. The results support the link between R/S and SOC and point to different religious/spiritual pathways to a strong SOC that are influenced by individual and cultural factors (64).

Individuals who utilize Ayurveda services mostly belong to the Society’s Leading Milieu (35.5%) with basic values being shaped by either tradition, modernization or re-orientation (69). At the same time, it is noteworthy that Ayurveda utilization can be found across all milieus, with a relatively consistent percentage ranging from 9 to 13.5%. However, the Traditional and Precarious Milieus may be apparent as individuals into whose lives Ayurveda has not yet made significant inroads (44). While milieu-concepts might have been a useful tool for understanding social groups, there could be valid concerns regarding their relevance and potential drawbacks. One of these concerns is the risk of oversimplification and stereotyping, as milieus tend to categorize individuals based on specific characteristics or behaviors (70). India’s pluralistic healthcare system does not explicitly promote TCIM, but the various traditional health systems covered by the Ministry of AYUSH (Ayurveda, Yoga and Naturopathy, Unani, Siddha, Sowa Rigpa and Homeopathy) (71). Ayurveda is widespread in India as a folk medicine and is practiced alongside Western medicine with diverse Ayurvedic trends and currents (18, 72). Ayurveda also faces “medicalisation,” meaning standardization, professionalization and pharmaceuticalization, which may impact the education, knowledge, practice and narrow the holistic view of traditional Ayurveda approaches (73, 74). The Ministry of AYUSH seeks to integrate diverse local health traditions, as well as traditional and complementary medicine into a modern healthcare system (12, 75). The European research network CAMbrella for complementary and alternative medicine (CAM) that operated between 2010 and 2012 and aimed to assess the situation of CAM in Europe, addressed that more research is needed and the integration of evidence-based or -informed CAM treatments into the Western healthcare systems. The findings emphasize the high demand for CAM, its heterogeneity, and the challenges in evaluating its effects due to insufficient integration and lack of validated data. Traditional Indian medicine is only mentioned in passing in one work package to provide a global perspective (76, 77).

The World Health Organization (WHO) is actively promoting the integration of traditional medicine into global health systems, emphasizing evidence-based and scientifically validated methods to guarantee the safety, qualification, and effectiveness of traditional, complementary, and integrative medicine (TCIM) services (20). Key points of the WHO vision include the development of norms and standards, the use of data and analysis to shape policies and regulatory frameworks, the promotion of sustainability and the integration of TCIM. This vision could be an impulse that could trigger corresponding measures at legal and political level for Ayurveda in Germany and Europe (20).

### 4.3 Further research

More high-quality data are needed to gain a deeper understanding of the use and impact of Ayurveda in Germany, given its growing popularity and potential to complement modern medicine. Furthermore, differentiation between therapy and wellness, and health policy measures are needed to establish scientific credibility, translate Ayurvedic concepts into mainstream western medical thinking and provide access to a wider audience. In addition, potential risks and side effects of Ayurveda should be investigated with adequate methodology. In addition, further methodologically high-quality studies are required to evaluate efficacy and effectiveness of Ayurveda in and outside its countries of origin.

## 5 Conclusion

Study results show that around one in 10 people in Germany has already used Ayurveda and around a third believes in its therapeutic potential. Ayurveda is mainly used by well-educated, higher-income women, typically between 20 and 40 years of age with interest in (vegetarian/vegan) nutrition and with a rather modern milieu-orientation. The perception of Ayurveda’s potential therapeutic benefits is influenced by factors such as a positive attitude toward TCIM and is associated with spirituality. Because Ayurvedic therapies are often not evidence-based, there is an urgent need to perform high quality randomized controlled trials to investigate potential effects and safety of Ayurveda and how evidence-based Ayurveda treatments can be integrated into the German healthcare system.

## Data availability statement

The raw data supporting the conclusions of this article will be made available by the authors, without undue reservation.

## Ethics statement

The studies involving humans were approved by Ethics Committee of Charité – Universitätsmedizin Berlin. The studies were conducted in accordance with the local legislation and institutional requirements. The participants provided their written informed consent to participate in this study.

## Author contributions

JS: Writing – original draft. MJ: Funding acquisition, Project administration, Visualization, Writing – review & editing. AM: Supervision, Writing – review & editing. ES: Writing – review & editing. MO: Writing – review & editing. MS: Writing – review & editing. BB: Supervision, Writing – review & editing. MW: Data curation, Formal analysis, Software, Writing – review & editing. CK: Conceptualization, Funding acquisition, Project administration, Supervision, Writing – review & editing.

## In memoriam

The authors dedicate this article to Boike Rehbein (†11.6.2022). Boike Rehbein was involved in the planning and initiation of the project and sadly passed away during the project.
