# Supplementary material for: Wellness or medicine? Use and perception of Ayurveda in Germany: data from an online-representative cross-sectional study
Source: Front Med (Lausanne). 2024 May 22;11:1408609. doi: 10.3389/fmed.2024.1408609 (PMC11150709; doi:10.3389/fmed.2024.1408609)
Supplement: Supplementary file 1 [file Data_Sheet_1.PDF]

Supplementary Material

Supplementary Material, Figure 1: Do you think Ayurveda has any medical therapeutic benefits? Ayurveda vs Non-Ayurveda Patient

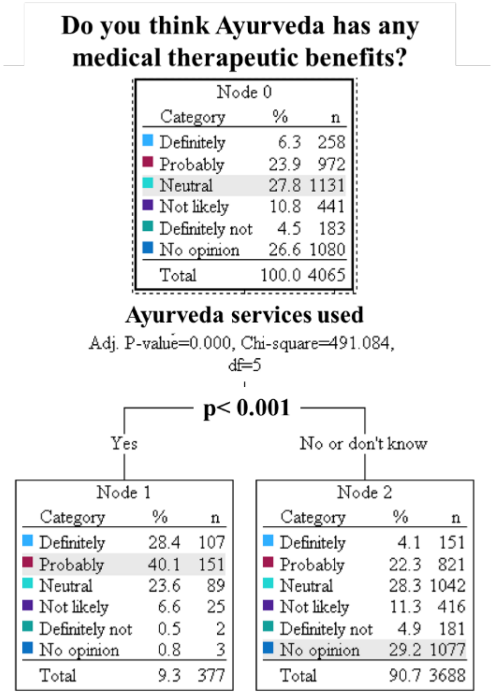

Supplementary Material, Figure 2 (a/b/c) Decision tree for the therapeutic benefits of Ayurveda.

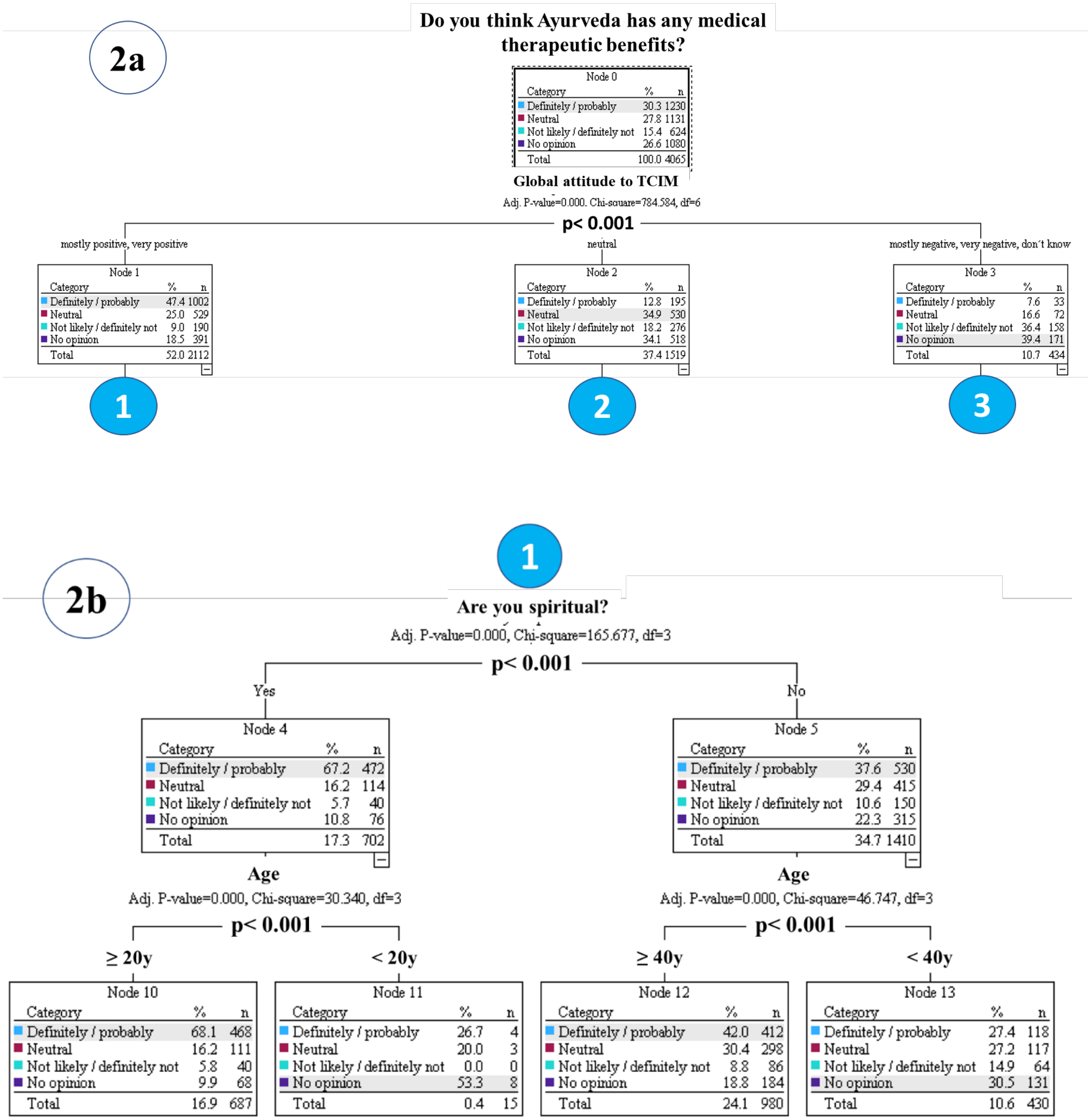

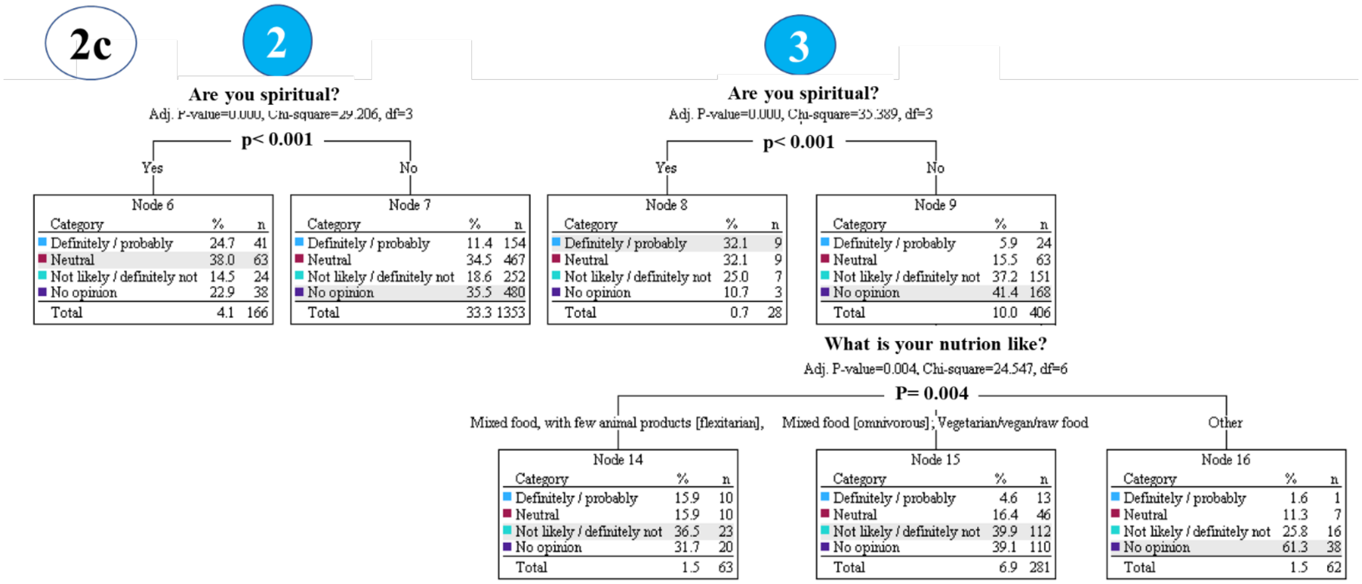

Supplementary Material, Figure 3: Would you describe yourself as spiritual? (n=4065)

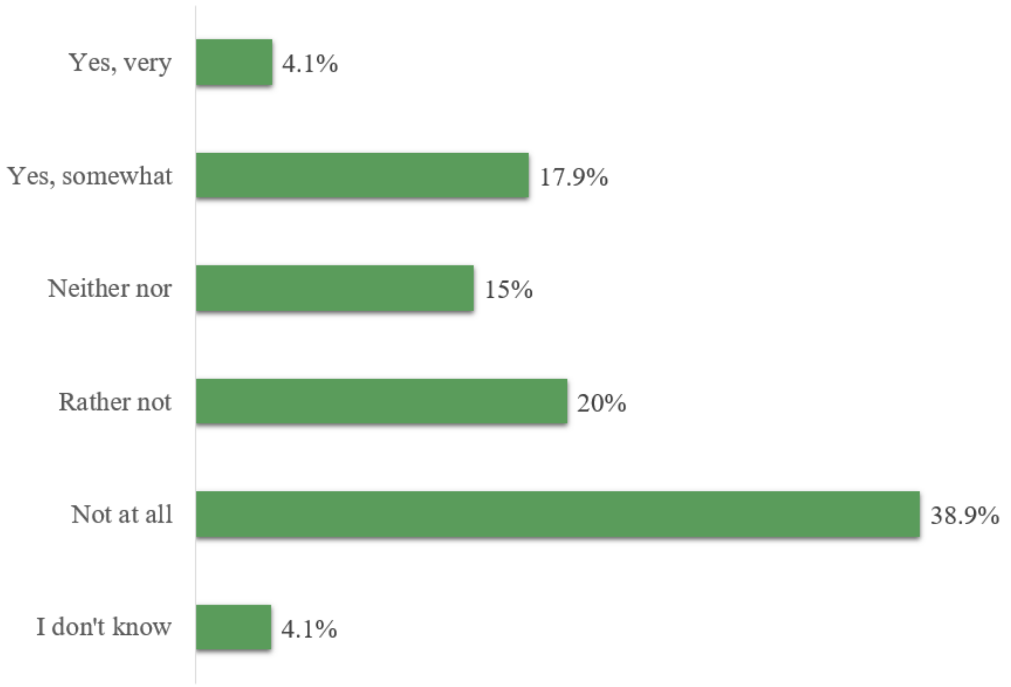

Supplementary Material, Figure 4: Comparison of religious status for Ayurveda users and Ayurveda non-users.

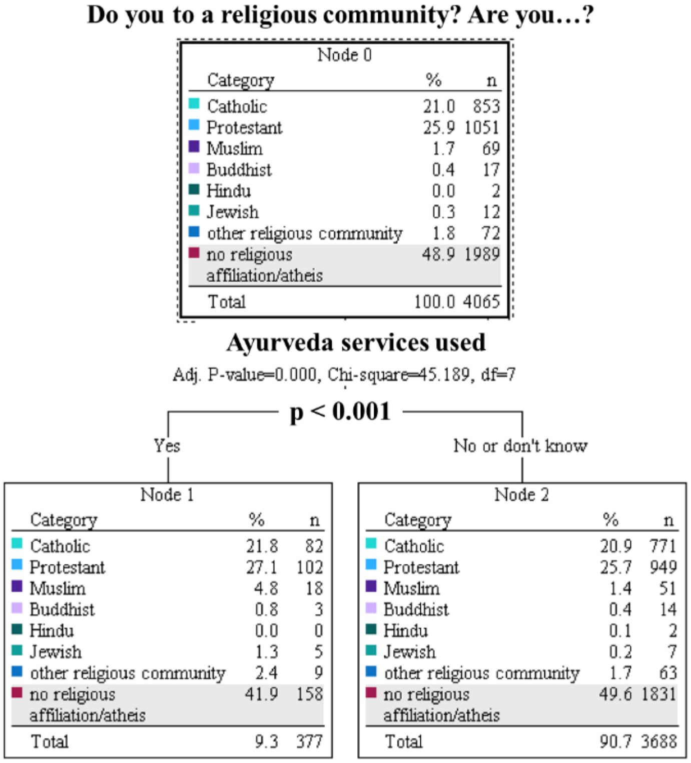

Supplementary Material, Figure 5: Description of subjects with or without Ayurveda services by basic parameters

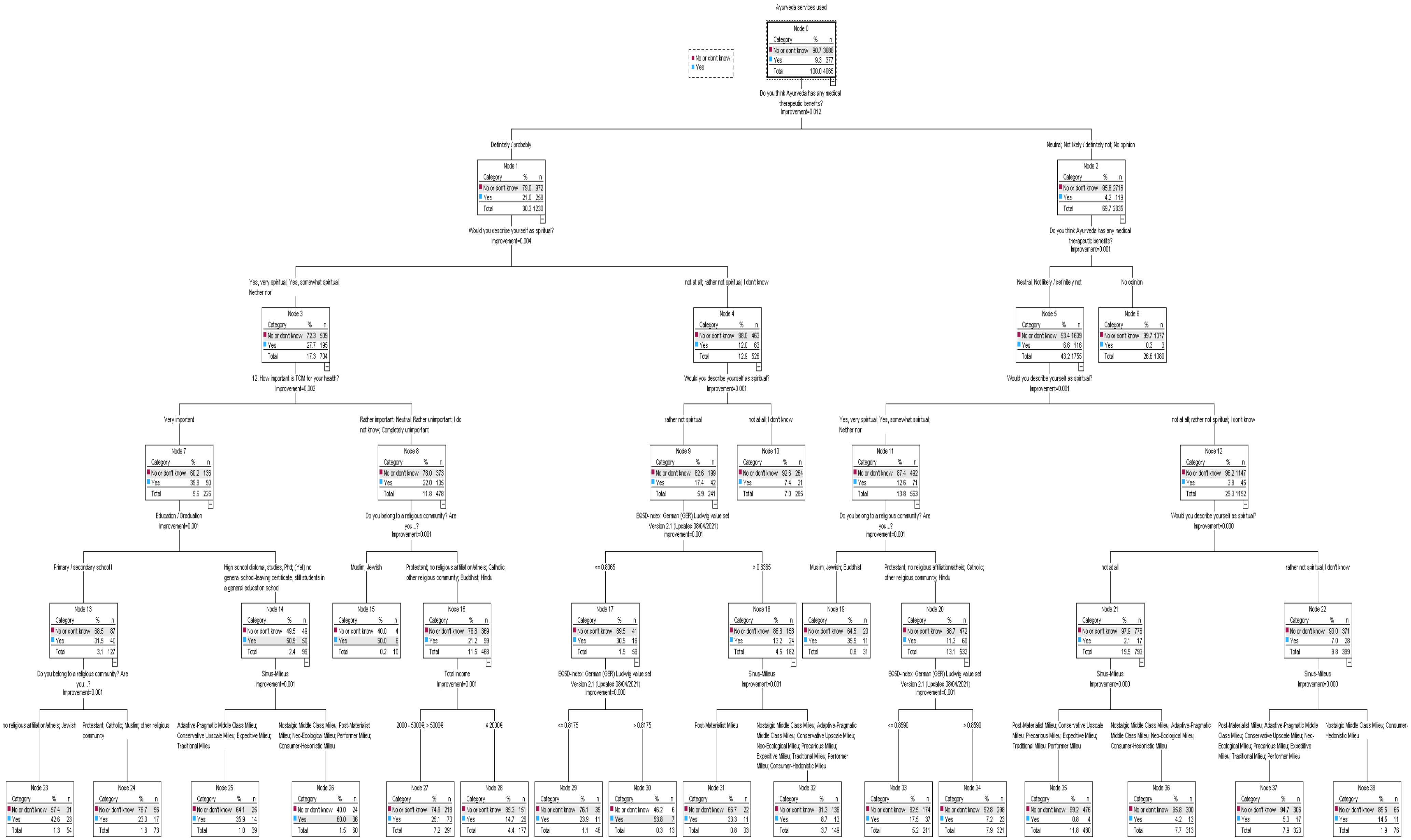

Supplementary Material, Figure 6: The role of healthy nutrition depending on the use of Ayurvedic services

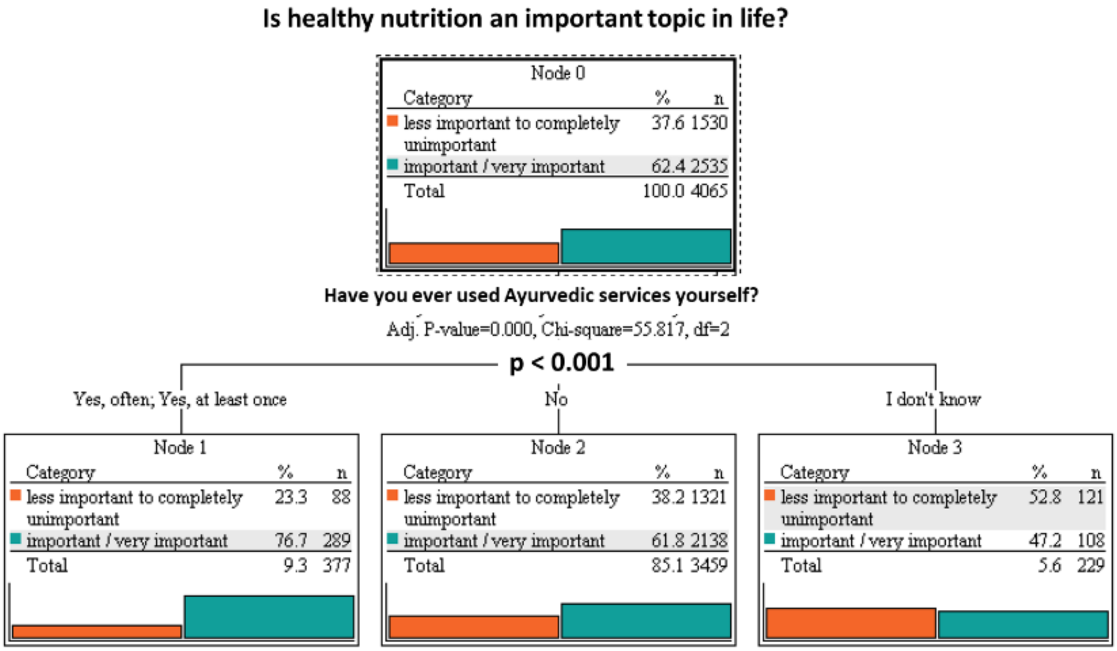

Supplementary Material, Figure 7: Choice of diet depending on the use of Ayurvedic services

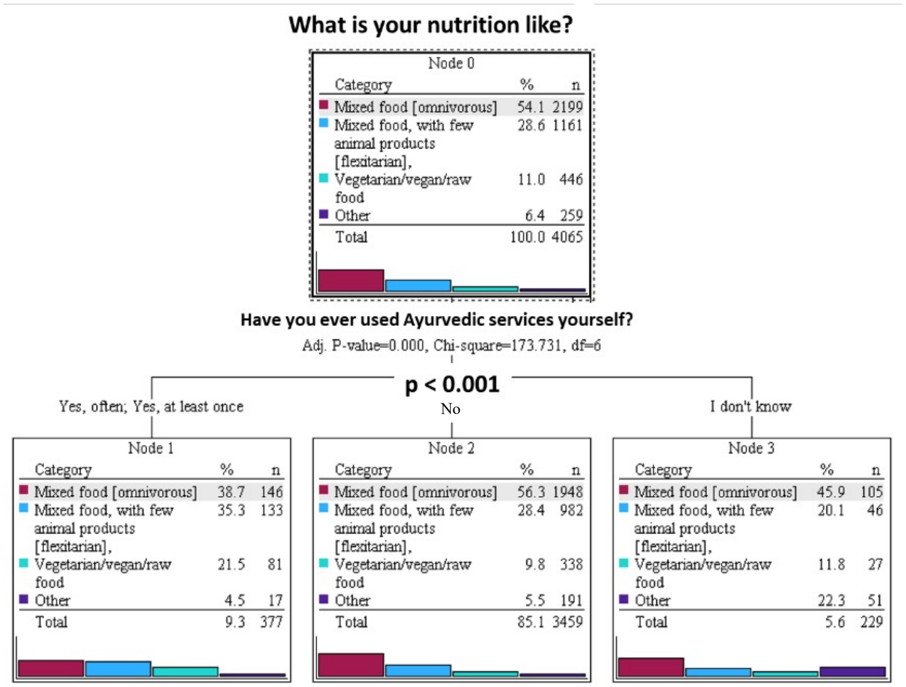

No

Supplementary Material 8: Questionnaire Ayurveda module

1. I associate Ayurveda first and foremost with

O	Indian Medicine
O	Nutrition
O	Spices
O	Massage
O	Wellness
O	Esotericism
O	Spirituality
O	Other: [Free text]
O	Nothing at all

2. Have you ever used Ayurveda services yourself?

O	Yes, more than once
O	Yes, at least once
O	No
O	Don’t know

3. Which Ayurveda service have you taken advantage of? Multiple answers possible

O	Advice on Ayurveda nutrition
O	Advice on Ayurveda lifestyle
O	Medical Ayurveda treatment
O	Non-medical Ayurveda treatment (e.g. by alternative practitioner, physiotherapist)
O	Ayurveda products (e.g. food, cosmetics, food supplements, medicines, food)
O	Ayurveda training
O	Infotainment (television, cinema, internet)
O	Other: [Free text]

4. Where have you taken advantage of Ayurveda services? Multiple answers possible

O	Outpatient practice (Physician, alternative practitioners)
O	Wellness center / Spa
O	Hotel
O	Hospital
O	Educational institution
O	Online
O	Other:

5. Do you believe that Ayurveda has a medical-therapeutic benefit?

O	Definitely
O	Probably
O	Neutral
O	Not likely
O	Definitely not
O	No Opinion

6. Would you describe yourself as spiritual?

O	Yes, very
O	Yes, something
O	Neither
O	Rather not
O	Not at all
O	Don't know
